# Severe Bradycardia Prior to Coronary Artery Bypass Graft Surgery: A Case Report

**Published:** 2018-07

**Authors:** Amer Harky, Mohamad Bashir, Ciaran Grafton-Clarke, Martin Lees, Sarah Fendius, Neil Roberts

**Affiliations:** 1 *Department of Cardiothoracic Surgery, Barts Heart Centre, St Bartholomew's Hospital, London, UK. *; 2 *Department of Vascular Surgery, Countess of Chester Hospital, Chester, UK.*; 3 *School of Medicine, University of Liverpool, Cedar House, Liverpool, UK.*; 4 *Department of Perioperative Medicine, Barts Heart Centre, St. Bartholomew’s Hospital, London, UK.*

**Keywords:** *Cardiac conduction system disease*, *Coronary artery bypass*, *Sick sinus syndrome*, *Arrhythmia, cardiac*

## Abstract

Intraventricular conduction abnormalities following cardiac surgery have been thoroughly described, especially after valvular surgery. It is also widely known that several anesthetic factors can cause autonomic disturbances resulting in the unmasking of sinus node dysfunction, significant bradycardia, and cardiovascular collapse during the intraoperative period. However, little is known about asymptomatic episodes, especially those occurring prior to coronary artery bypass grafting (CABG). We report a rare occurrence of an intraventricular conduction defect that presented in an asymptomatic patient following non–ST-elevation myocardial infarction prior to urgent CABG. Our patient presented with sudden-onset chest pain, and following coronary angiography he was found to have triple-vessel coronary disease. During anesthetic induction for inpatient CABG surgery, he developed episodes of acute sinus tachy-brady episodes, requiring a stat dose of adrenaline to maintain the heart rate prior to the establishment of cardiopulmonary bypass. The arrhythmia persisted postoperatively, necessitating the insertion of a permanent dual-chamber pacemaker for complete heart block. The patient was later discharged without further complications, and upon follow-up 12 months later, he remains in good health.

## Introduction

Sick sinus syndrome is a term used to describe the inability of the sinoatrial node to generate a heart rate that meets the physiologic needs of an individual.^1^ The sick sinus spectrum of heart rhythms includes a subgroup of alternating atrial bradycardia with episodes of atrial tachyarrhythmia. Tachycardia-bradycardia is an electrophysiologically and therapeutically interesting clinical entity that can complicate the effective management of acute myocardial infarcts.^2^ This can induce sinus arrest, sinus node exit block, sinus bradycardia, and sinus tachycardia, and is also associated with paroxysmal supraventricular tachycardia and atrial fibrillation.^3^ Prior to surgery, patients with this arrhythmia require special care due to the risk of cardiac failure, embolization, and sudden cardiac death.^3^ Whilst this clinical syndrome often presents with hemodynamic instability, syncope, and features of cardiac failure, it is not uncommon for patients to be asymptomatic.^4^ The treatment of choice in these patients is the placement of a pacemaker, namely atrial or dual chamber devices, which have been demonstrated to reduce the incidence of atrial fibrillation, thromboembolic events, and mortality.^5^

## Case Report

**Figure 1:**
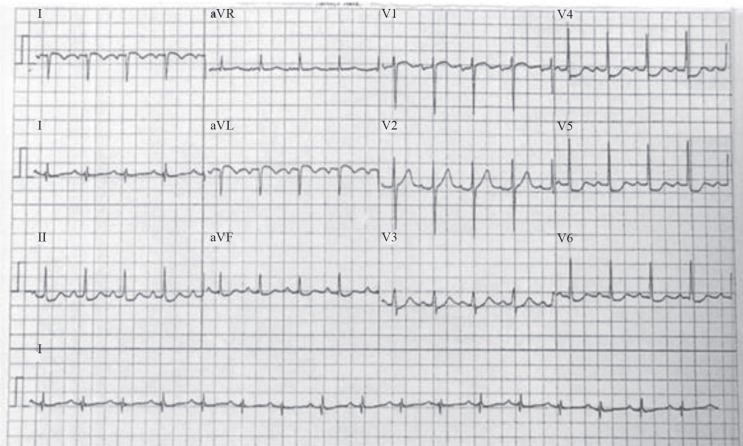
Preoperative electrocardiogram of the patient, showing normal sinus rhythm with inferolateral ST depression.

We present the case of a 61-year-old man who was admitted to our center with sudden-onset chest pain. The patient was diagnosed with non–ST-elevation myocardial infarction. His electrocardiogram (ECG) showed normal sinus rhythm with inferolateral ST-segment depression (Figure 1). The patient’s past medical history included controlled hypertension, non–insulin-dependent diabetes mellitus, peripheral vascular disease, hypercholesterolemia, iron deficiency anemia, hepatitis B, and being an ex-smoker. 

The patient’s immediate angiogram revealed a right-dominant heart with severe distal left main stem disease and an 80% occlusion. The left anterior descending artery (LAD) was diffusely irregular with severe mid-vessel disease. It was noted that he had a high diagonal artery, in which the proximal portion was severely diseased. The left circumflex artery was both severely and diffusely diseased and very tortious. The right coronary artery was diffusely diseased. Due to the presence of diffuse multi-vessel involvement, the patient was deemed suitable for inpatient coronary artery bypass grafting surgery (CABG). He was medically stabilized and optimized for surgery.

Seven days following his initial presentation, the patient underwent CABG. On the induction of anesthesia and during the insertion of the central venous line into the right internal jugular vein, the patient developed an acute episode of sinus tachycardia followed by sinus bradycardia of 40 beats per minute. A bolus dose of adrenaline (5 μg IV) was given to maintain his heart rate. The preliminary impression was that the patient had sustained an infarct affecting the atrioventricular conduction system. Cardiopulmonary bypass (CPB) was instituted immediately, and the patient’s status was stabilized. 

CABG was performed on the patient utilizing the left internal mammary artery to the LAD and the saphenous venous graft to the obtuse marginal and the posterior descending artery. The CPB time was 77 minutes, and the aortic cross-clamp time was 35 minutes. He was weaned off CPB support in a state of sinus bradycardia. Two epicardial pacing wires were placed, and the patient was externally paced. 

Three days following the CABG surgery, the patient became hemodynamically unstable with alternate episodes of tachy-brady arrhythmia concomitant with atrial fibrillation. For rate control, he was loaded with intravenous amiodarone. His status necessitated inotropic support with noradrenaline to maintain an adequate blood pressure and urine output. Bedside transesophageal echocardiography ruled out cardiac tamponade and demonstrated no evidence of a left atrial thrombus.

**Figure 2:**
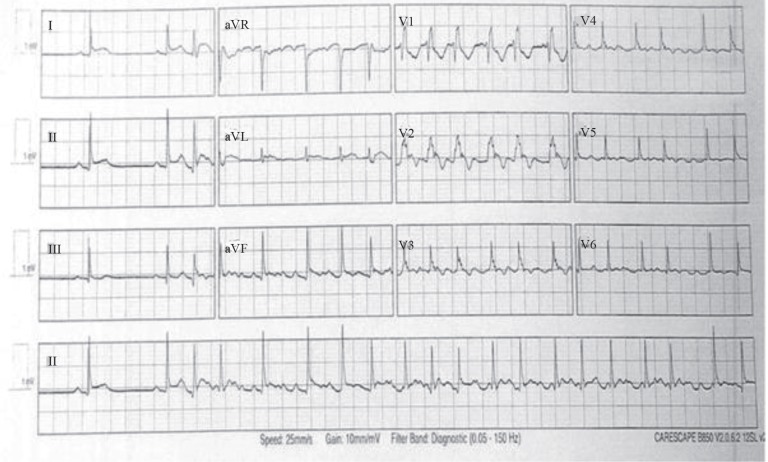
Postoperative electrocardiogram, showing electrical pauses as an indication for the insertion of a permanent pacemaker.

Subsequently, an urgent consultation with our local electrophysiology team was sought. It was noted that the tachy-brady arrhythmia included multiple electrical pauses (Figure 2) alternating with episodes of atrial fibrillation. A decision was made to insert a dual-chamber permanent pacemaker (PPM). Following the insertion of the PPM, the patient experienced self-limiting episodes of atrial fibrillation associated with a fast-ventricular response rate; he was commenced on beta-blockers. Once the heart rate was controlled, he was discharged from the intensive care unit to a normal ward and made a complete recovery.

## Discussion

Electrical conduction abnormalities are well-recognized complications of acute myocardial infarction.^6^ They are caused by either autonomic imbalance or ischemia and subsequent necrosis of the conduction system. The most common clinical presentation is bradycardia, which may or may not be symptomatic. Complete heart block with a slow escape rhythm, if not detected and treated promptly, is a potentially fatal event.

Anesthesia and associated surgical maneuvers can lead to autonomic imbalance, which may unveil an undiagnosed sick sinus syndrome.^7^ The resultant dysrhythmias can be resistant to conventional pharmacological treatment. In patients with a known diagnosis of sick sinus who are asymptomatic, preoperative insertion of a temporary pacemaker prior to anesthetic induction should be contemplated. 

Preoperative, intraoperative, and postoperative variables which may play a role in the development of ventricular conduction defects and atrial fibrillation following CABG have been previously described in the literature. Caretta et al^8^ evaluated 236 consecutive patients who sustained ventricular conduction defects and atrial fibrillation postoperatively and reported an incidence of 15.5% amongst their patient cohort. In their analysis, it was demonstrated that left main disease and right coronary artery occlusion associated with significant stenosis of the proximal LAD were amongst the factors that could lead to conduction abnormalities. A further contributory factor that the authors identified was the aortic cross-clamp time.

Because of these risks, it is well known to practicing cardiac surgeons that atrial fibrillation can develop postoperatively,^9^ with the link between ischemic injuries sustained by increasing durations of cardioplegic arrest seeming to play a key role in such incidence. Non-homogeneous cardioplegia delivery to critical areas of the myocardium, particularly to the specialized conducting system, may cause ventricular conduction defects after CABG. 

Passman et al^10^ described a model whereby the incidence of atrial fibrillation post cardiac surgery could potentially be quantified and predicted. The occurrence of conduction abnormalities may be related to advanced age (>65 y), PR intervals of at least 180 ms, and P-wave durations in lead V_1_ of at least 110 ms. Such peculiarities could lead to prevention protocols to avert patients from sustaining atrial fibrillation post cardiac surgery, thus benefiting patient health as well as reducing the duration of hospital stay and cost.

In this case report, we demonstrated that an intraventricular conduction defect occurred on the induction of anesthesia in an otherwise asymptomatic patient with normal sinus rhythm on ECG preoperatively. Bradycardia is associated with agents such as vecuronium, alfentanil, and propofol. Pre-treatment with anti-cholinergic agents may reduce incidences of conduction abnormalities in healthy individuals. Hence, bradycardia under anesthesia should not be assumed to be iatrogenic as the cause may be sick sinus syndrome.^11^ Bearing this in mind, we deemed it necessary to expedite the patient to the operating room and to commence CPB immediately with a view to averting further injury to the heart and avoiding peri-arrest episodes. Despite these measures to correct the underlying ischemic causes of the acute intraventricular stunning of the conduction system, the patient continued to display signs of persistent tachyarrhythmia, even after PPM insertion. 

## Conclusion

This is a unique experience that opens the scope for further investigations to predict the occurrence of asymptomatic intraventricular conduction abnormalities prior to CABG and to identify any correctable factors that could be addressed to protect these patients. 

## Notes:

***This paper should be cited as: ***Harky A, Bashir M, Grafton-Clarke C, Lees M, Fendius S, Roberts N. Severe Bradycardia Prior to Coronary Artery Bypass Graft Surgery: A Case Report. J Teh Univ Heart Ctr 2018;13(3):136-139.
